# Development of Volatile Compounds in Raw Fermented Sausages with Reduced Nitrogen Compounds—The Effect of Tomato Pomace Addition

**DOI:** 10.3390/molecules29245826

**Published:** 2024-12-10

**Authors:** Patrycja Skwarek, Jose M. Lorenzo, Laura Purriños, Małgorzata Karwowska

**Affiliations:** 1 Sub-Department of Meat Technology and Food Quality, Department of Animal Food Technology, University of Life Sciences in Lublin, Skromna 8, 20-704 Lublin, Poland; patrycja.skwarek@up.lublin.pl; 2 Centro Tecnológico de la Carne de Galicia, Rúa Galicia N° 4, Parque Tecnológico de Galicia, San Cibrao das Viñas, 32900 Ourense, Spain; jmlorenzo@ceteca.net (J.M.L.); laurapurrinos@ceteca.net (L.P.); 3 Area de Tecnología de los Alimentos, Facultad de Ciencias de Ourense, Universidad de Vigo, 32004 Ourense, Spain

**Keywords:** raw fermented sausages, tomato pomace, volatile compounds, bioactive compounds, sensory analysis

## Abstract

The aim of this study was to assess the impact of tomato pomace (TP) on the content of volatile compounds and L-carnitine and the sensory characteristics of raw fermented sausages produced with reduced nitrite. The produced sausages were divided into three experimental groups: control sample, sample with 1.5% addition of freeze-dried tomato pomace, and sample with 2.5% addition of TP. The results showed that the addition of tomato pomace significantly affected the quality of raw fermented sausages. Lower L-carnitine content was observed in samples with TP. The main groups of volatile compounds identified in fermented sausages were alcohols, aldehydes, hydrocarbons, and ketones. The addition of TP influenced the smell and taste of the sausages, which were characterized by a more intense tomato taste and smell and more intense red color compared to the control sample. Despite the influence of TP addition on some sensory features, the products were characterized by a high score of overall quality of over 7 c.u. on a 10-point scale, similar to the control sausage.

## 1. Introduction

Due to insufficient storage stability, meat had to be subjected to various methods of preservation in the past. One strategy was to grind the meat with salt and spices and to reduce the water content by drying. In this way, fermented sausages were created, which are still valuable traditional meat products today. Nowadays, many different types of sausages are produced using very different recipes and production processes. Traditional fermented sausages were considered healthy and safe food. Recently, however, eating these types of meat products has been linked to health risks due to the presence of nitrites/nitrates and nitrosamines [1]. Nitrites are widely used in meat products due to their beneficial effects on food quality and safety. They effectively prevent the growth of pathogens, including *Clostridium botulinum*; impart a characteristic color to the meat; and limit lipid oxidation processes, extending shelf life and preventing rancidity. At the same time, their use raises concerns about the formation of N-nitroso compounds, including nitrosamines, which have carcinogenic properties [2,3]. In this context, the meat industry is under pressure to search for natural and safe ingredients that would replace the use of synthetic additives in line with “clean label” trend. Therefore, replacing synthetic additives with plant raw materials in meat product recipes could be an interesting and innovative alternative [4]. One example is tomato pomace, which is a byproduct of tomatoes and is rich in valuable nutrients. It contains significant amounts of dietary fiber, pectin, lycopene, polyphenols, and vitamins, which makes it an attractive addition in various branches of the food industry. It has antioxidant properties and the ability to neutralize free radicals thanks to high contents of lycopene and polyphenols, which help protect cells against oxidative stress and may support the prevention of lifestyle diseases. Additionally, thanks to the content of lycopene, it improves the intensity of the red color of food products, such as raw fermented sausages. Moreover, it has antibacterial properties, which result from the presence of phenolic acids and flavonoids [5]. Therefore, this natural ingredient has great potential as an additive to meat products, which is also confirmed by our previous research results [6]. However, there is a lack of research assessing the impact of this plant raw material on the content of volatile organic compounds (VOCs) and bioactive compounds (L-carnitine) and the sensory parameters in fermented meat products. It is well known that volatile compounds of fermented products are formed as a result of numerous and complex reactions. Most of these reactions occur during the ripening phase and result mainly from carbohydrate fermentation and lipolytic and proteolytic processes [7].

Biochemical reactions, including lipolysis and proteolysis, which occur during the production of raw fermented sausages, are also necessary for the development of volatile compounds, as they provide precursors, free amino acids, and fatty acids, which are broken down in later stages to produce aromatic compounds [8]. The studies conducted so far contain little information regarding the impact of a reduction in nitrogen compounds on the development of specific flavor in meat products. A common trend in the study of volatile compounds is to analyze changes in their profile due to various factors, including reduction/elimination of nitrites, addition of plant raw materials, and identification of individual groups of volatile compounds [9]. Red meat, including pork, is also a rich source of many valuable endogenous compounds, such as proteins of high biological value, essential amino acids, microelements, and bioactive compounds (carnitine, conjugated linoleic acid (CLA), endogenous antioxidants, and creatine), which have antioxidant and health-promoting properties, including immunomodulating and protective effects against oxidative stress [10,11]. However, as is well known, oxidative reactions of both proteins and fats affect the quality of meat products, including sensory quality (taste, color, and texture). In addition, they negatively affect color, which in turn affects consumer acceptance, resulting in product rejection, as consumers often associate color with freshness and quality of meat [12]. Therefore, the important challenge associated with tomato byproducts is to maintain the sensory properties of meat products that can be accepted by consumers. As is known, the presence of L-carnitine in fermented products may indirectly affect the activity of microorganisms and the profile of metabolites. Lactic acid bacteria, often used in fermentation, can modify the metabolism of lipids and proteins, which later influences the formation of specific volatile compounds. Changes in lipid metabolism caused by the activity of L-carnitine may also lead to differences in the aromatic profile during meat maturation [13,14].

In this context, the main aim of this work was to evaluate the effect of tomato pomace powder on the development of volatile compounds, L-carnitine content, and sensory profile of raw fermented sausages produced with reduced nitrogen compounds. 

## 2. Results

### 2.1. Volatile Compounds

**Figure 1:**
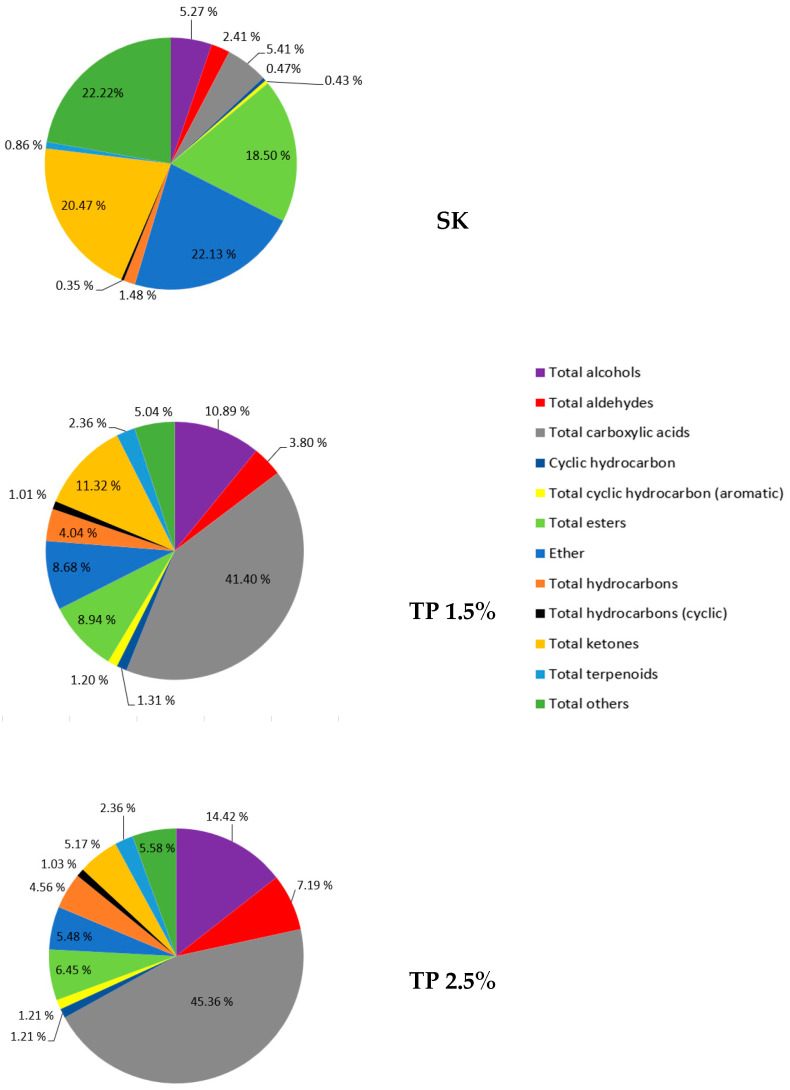
Volatile families in raw fermented sausages. SK—control sample; TP 1.5%—sample with 1.5% addition of tomato pomace; TP 2.5%—sample with 2.5% addition of tomato pomace.

Table 1 presents the effect of tomato pomace addition on the content of volatile compounds in raw fermented sausages. A total of 62 volatile compounds were identified in the experimental meat products after the production process. They were classified according to their functional group: 11 alcohols, 9 aldehydes, 3 carboxylic acids, 1 cyclic hydrocarbon, 2 cyclic hydrocarbons (aromatic), 7 esters, 1 ether, 8 hydrocarbons, 2 hydrocarbons (cyclic), 7 ketones, 8 terpenoids, and 8 compounds classified as “other”. The main groups were alcohols, aldehydes, hydrocarbons, and ketones. Statistically significant differences (*p* < 0.05) were observed between treatments in the content of volatile compounds from the group of alcohols, aldehydes, carboxylic acids, esters, and ketones. Total compounds did not differ statistically significantly between the samples. However, SK sausage presented the greatest amount of total volatile substances, mainly contributed by esters, ether, ketones, and others (Figure 1). On the other hand, carboxylic acids represented more than 40% of the total compounds in the samples with TP.

### 2.2. L-Carnitine

The use of freeze-dried tomato pomace had a statistically significant effect on the level of L-carnitine in raw fermented sausage (Table 2). The control sample was characterized by a higher content of this bioactive compound compared to the samples with tomato pomace addition. The content of L-carnitine in the experimental sausages ranged from 1.39 to 2.51 mg 100 g^−1^.

### 2.3. Sensory Parameters

Table 3 presents the results of the sensory evaluation of raw fermented sausages with the addition of tomato pomace. In general, the addition of tomato pomace in the amounts of 1.5% (TP 1.5%) and 2.5% (TP 2.5%) presented similar overall quality obtained for the control sausage (SK). No significant differences among treatments were indicated in case of most of the evaluated parameters, such as color of fat, juiciness, hardness, intensity of other odors and flavors, intensity of saltiness, and sweet flavor. Statistically significant differences were shown between the sausage treatments in terms of color, intensity of meat, and tomato flavor and aroma. The addition of tomato pomace caused significantly higher values of the evaluation of meat color intensity by cross-section. The meat aroma intensity decreased, while the tomato aroma intensity increased significantly. Interestingly, the addition of tomato pomace in amounts of both 1.5 and 2.5% affected the intensity of tomato aroma to a similar extent.

## 3. Discussion

The specific taste of meat products is determined by mixtures of volatile compounds. The share of individual volatile compounds in the creation of a characteristic taste varies. Only a small part of this huge number of volatile compounds present in food products contributes to the development of taste, which is created as a result of multidirectional reactions occurring between non-volatile precursors contained in raw meat [15]. Volatile compounds are created as a result of many factors such as the Maillard reaction, lipid oxidation, interactions between Maillard reaction products and lipid oxidation products, as well as thiamine degradation. The taste is determined by many factors related to the raw material (e.g., breed, sex, diet, and age of the animal; slaughter conditions and process; time and conditions of meat storage; type of muscles), additives used, and the technological process [16].

Alcohols play a key role in the development of the aroma of raw fermented meat products. In this study, no statistically significant differences were found in the total alcohol content between the tested samples. They contained significant amounts of 1-pentanol (with a sweet, balsamic aroma), 1-butanol, and 3-methyl (fruity, whiskey), which are formed as a result of fat oxidation and degradation of amino acids. Comparing the obtained results, it was found that the content of volatile compounds was higher in comparison to those obtained for raw fermented sausages with the addition of chia and black cumin seeds [17]. In this study, volatile compounds (belonging to the group of alcohols) from tomatoes were also identified, which included 2-heptanol, 6-hepten-1-ol, 2-methyl, benzyl alcohol, phenol, and 2-methoxy [18,19]. The content of these volatile compounds was statistically significantly higher compared to the control sample and increased with the increase in the addition of tomato pomace. 2-heptanol and 6-hepten-1-ol give a fresh, fruity scent that can enrich the aroma of the sausage, giving it a more complex note. 2-methyl, benzyl alcohol, in turn, introduces floral, sweet accents that improve the overall sensory experience. Phenol, 2-methoxy in small amounts can add spicy, slightly smoky fragrance notes that are valued in this type of meat product. Therefore, tomato pomace affects the aromatic profile of fermented sausages, enriching them with new, fresh notes, but high concentrations of these compounds may lead to the aroma of the sausage being dominated by an intense fruity, floral, or chemical smell, making the product less attractive. Proper control over the amount of tomato pomace added allows for obtaining an optimal aromatic profile while avoiding excessive impact on the final taste of the product [20].

In fermented sausages, the main aromatic compounds are aldehydes, including hexanal, pentanal, octanal, and 2-nonenal (grass and green odors) [21]. Lactic acid bacteria also produce aldehydes such as 2-methyl- and 3-methyl-butanal [22]. Higher concentrations of the mentioned aldehydes were found in sausages with 2.5% tomato pomace, which suggests that their addition accelerates the degradation of amino acids. Benzaldehyde and benzeneacetaldehyde improve the taste, and 3-methyl butanal is one of the strongest aromatic compounds in fermented sausages [23,24]. These volatile compounds are present in large quantities in tomatoes. As a result, it had a positive effect on the taste and smell of sausages, which was confirmed by the results of the organoleptic analysis [25]. It should be noted, however, that these compounds can enhance the flavor of sausages, giving them a more intense, fresh, and slightly tangy character. However, their excess may lead to the flavor of the sausage being dominated by plant-like notes or overly intense acidity, which is not always desirable. Moreover, the highest value among all aldehydes was achieved by hexanal, produced as a result of the oxidation of linoleic acid, which is an indicator of meat quality and oxidative stability. In tests with the addition of tomato pomace, a lower content of this compound was found, which proves the pomace’s antioxidant effect [26]. It can therefore be concluded that tomato pomace, rich in natural antioxidants, can act as a pro-oxidant, reducing the content of aldehydes responsible for fat oxidation. These results are consistent with studies by other authors [17,27]. This was most likely due to the fact that the authors tested the same type of meat product, and as we know, as a result of fermentation and chemical reactions occurring during this process, characteristic groups of aldehydes are formed.

Carboxylic acids were another group of volatile compounds found in the fermented sausages assessed. The origin of carboxylic acids results mainly from the oxidation of unsaturated fatty acids and the hydrolysis of phospholipids and triglycerides [28]. The most important aromatic compounds from the group of carboxylic acids that are formed in fermented products include acetic acid, butanoic acid, and 3-methylbutanoic acid (cheese notes) [21]. This was also confirmed in our own studies, which found only three compounds belonging to this family. The control sample was characterized by their lowest content of these compounds. Both butanoic acid (unpleasant fermented, resembling rotten cheese) and acetic acid (pungent, sour) are products that are formed as a result of the fermentation of carbohydrates produced by lactic acid bacteria. They are closely related to the typical aroma of fermented sausages, because they emit a very strong smell [24]. In the current study, the meat products with tomato pomace contained more of these acids, which was consistent with the observations of other authors who studied sausages fermented with the addition of various seeds [17]. These compounds were present in similar amounts in all the experimental sausages (for acetic acid: from 2809.05 to 11,189.16 AU × 10^3^ g^−1^; for butanoic acid: from 1201.48 to 2767.75 AU × 10^3^ g^−1^). Taking into account the data on acetic acid, a larger amount of tomato pomace caused an increase in this substance, which is characterized by a very intense vinegar smell. This compound is also classified as a volatile compound originating from bacterial metabolism. It is known that lactic acid bacteria metabolize carbohydrates, reducing the pH drop as a result of the formation of lactic acid, which makes the taste of fermented meat products rather sour. Moreover, the addition of tomato pomace, which is characterized by high acidity, causes an even greater decrease in pH and, consequently, an increase in the content of acetic acid in the produced sausages, which also confirms our observations [29].

In the experimental sausages, seven esters were identified, formed through lipid autoxidation (hexanoic acid), carbohydrate fermentation (acetic acid, butanoic acid), and staphylococcal esterase activity (ethyl acetate). Ethyl lactate, a compound with a pop-corn, buttery, fruity, and floral aroma, was also found, confirming the presence of lactic acid bacteria involved in fermentation. The total ester content did not differ significantly (*p* > 0.05) between treatments. However, sausages with tomato pomace had higher levels of acetic acid, butanoic acid, and hexanoic acid, likely due to the higher carboxylic acid content in the pomace. Compared to the studies of other authors [17,30], the presence of methyl salicylate was found only in the present study. It is one of the most important volatile substances responsible for the taste of tomatoes [30]. As could be observed, only the control sample did not contain this compound, and with the increased concentration of tomato pomace, the sausages were characterized by a significantly higher level of this volatile compound. In the conducted studies, no statistically significant differences were observed in the content of total hydrocarbons and cyclic hydrocarbons. Samples with tomato pomace, however, contained larger amounts of n-hexane, butane, 2,2,3,3 tetramethyl, heptane, 2,2,4,6,6 pentamethyl, 1,3,5,7-cyclooctatetraene, cyclopropane, and propyl, and smaller amounts of octane. The aromatic compounds present in the sausages mainly came from lipid autoxidation: heptane, n-hexane, and pentane. It should be noted that branched hydrocarbons were the most abundant. A total of eight different compounds were identified, followed by two cyclic hydrocarbons. However, statistically significant differences were found in the quantity of individual volatile compounds from the ketone family between the tested groups of raw fermented sausages. The highest content of almost all ketones was characteristic of sausages with the addition of tomato pomace. It was noted that a larger share of this plant additive caused a significant increase (*p* < 0.05) in the content of these ketones. Two methyl ketones (5-hepten-2-one, 6-methyl, 2-nonanone) were detected in the produced sausages, which, as a result of the degradation of free fatty acids, contribute to the formation of the fatty aroma of meat products [31]. The largest amount of acetoine was identified in the tested meat products, which is produced mainly as a result of microbiological fermentation of carbohydrates with the participation of lactic acid bacteria [23]. Another byproduct of LAB metabolism is 2-butanone. Samples with tomato pomace were characterized by a higher content of this compound [32]. In the experimental sausages, the presence of compounds such as acetone, 1-hepten-3-one, 5-hepten-2-one, 6-methyl, acetophenone, and 2-nonanone was associated with the addition of tomato pomace. Their content increased with the increase in the concentration of the plant additive. Both 1-hepten-3-one and 5-hepten-2-one, 6-methyl are among the main volatile aromas of tomatoes, while 2-nonanone is characterized by a fruity smell and sweet taste [25,33].

The next group of compounds identified in the experimental sausages were terpenoids. Among the identified compounds from the terpenoid group were 3-carene and bicyclo[3.1.0]hex-2-ene, 4 methyl-1-(1-methylethyl) (beta-thujene). 3-Carene is a bicyclic monoterpene consisting of fused cyclohexene and cyclopropane rings. It is characterized by a sweet and pungent odor, best described as a combination of fir needles, musky earth, and damp forests [34]. β-thujene is also a bicyclic monoterpenoid that can be derived from various vegetable oils. It also occurs in relatively high concentrations in the essential oils of some plants. It is considered an important volatile compound, giving a woody character [35]. No statistically significant differences in the content of substances from the terpenoid group were found between the samples. In the category “other”, a total of eight volatile compounds were identified. The most abundant volatile compound in this category was 2-isobutylthiazole, and its content increased with the increase in the concentration of tomato pomace. It is a component characteristic of ripe tomatoes [36].

The consumer acceptability of fermented meat products is largely influenced by the final taste. The typical taste and smell of dried sausage cannot be attributed only to volatile substances, but to a large number of volatile and non-volatile compounds present in the product in appropriate proportions. Volatile compounds mainly affect the aroma of raw fermented sausage [37]. The fermentation process of sausages, apart from ensuring product safety and extending shelf life, is also aimed at imparting specific taste and smell characteristics. It should also be noted that reducing the nitrite content in raw fermented sausages could also have an impact on the content of volatile compounds and the final taste of the assessed product. Reducing the content of nitrogen compounds in this type of meat product may affect the activity of lactic acid bacteria, which may lead to a change in the profile of volatile compounds due to differences in bacterial metabolism. It may also influence the content of bioactive compounds, such as L-carnitine, which affects the metabolism of fats and proteins. This treatment may also affect the speed of sausage ripening and the speed of reactions, leading to the formation of volatile compounds. This may result in differences in the intensity of some scents, e.g., floral, fruity, or herbal. In this study, it was assumed that the addition of tomato pomace would increase the attractiveness of the meat product and thus additionally have a health-promoting effect. In general, all samples obtained good results in all assessed sensory parameters. Moreover, no significant differences were found in the values of fat color in cross-section, juiciness, hardness, intensity of other smells, salty, sweet, other taste, and overall quality, and therefore they were mostly comparable to the control sample. However, significant differences were observed between treatments in terms of meat color in cross-section, meat and tomato flavor, and aroma intensity. According to Domínguez et al. [38], the main volatile compounds influencing the aroma of the final product belong to different chemical families, but not all of them have the same importance in the overall perception of aroma. Alcohols have a significant effect on the aroma of dried fermented meats due to their low aroma threshold, as they participate in many metabolic pathways, including lipid oxidation, amino acid metabolism, and methyl ketone reduction [39]. Aldehydes, esters, and ketones are also among the most important groups of compounds derived from lipid degradation in meat products and thus shape the taste and aroma of the final product [40]. In this study, in raw fermented sausages, it was within these groups that the largest number of volatile compounds was identified, a large part of which were compounds derived from tomato pomace, influencing the taste and aroma of the produced raw fermented sausages.

Due to the content of natural pigments (carotenoids and polyphenols), color is one of the main sensory effects induced by tomato pomace. In general, the addition of TP to a food product causes a decrease in lightness (color value L*) while significantly increasing redness (color value a*) and yellowness (color value b*) [41,42]. This was confirmed in our studies, where the panelists noticed differences in the color of sausages with the addition of tomato pomace. Samples of TP 1.5% and TP 2.5% were characterized by a more intense red color. These were in line with the results obtained by other authors, who reported statistically significant differences (*p* < 0.05) in the color between samples of pork products with the addition of tomato paste [43]. The studies of Kim et al. [44] showed that the addition of tomato powder had no effect on the smell, taste, texture, or overall acceptability of pork sausages. However, this was contrary to the observations in the present study, in which the sensory panel team observed significant changes in the taste of sausages caused by the addition of tomato pomace compared to the control samples. The differences in the results could be due to the fact that different types of meat products were produced using different techniques, including cooking or heat treatment, which could mask the smell and taste of tomatoes in the final product. Our observations regarding the taste parameter were consistent with studies on the quality of beef hamburgers enriched with lycopene using dried tomato peel [45]. The researchers confirmed that meat products with the addition of dried tomato peel were characterized by a more intense tomato taste compared to the control sample.

In addition to chemical composition and sensory properties, the amount of bioactive compounds are very important in the development of new food products. L-Carnitine is a component necessary for energy production and lipid metabolism in many organs and tissues, such as skeletal muscle and the heart. Additionally, L-carnitine participates in the oxidation of fatty acids in peroxisomes, maintaining the CoA/acyl-CoA ratio in the cell, and the utilization of ketone bodies. L-carnitine deficiency can lead to metabolic disorders affecting muscle and heart function and manifest as myopathy or heart disease [46]. Animal products, especially meat, are a very valuable source of L-carnitine in the human diet, and its content depends on the type of product, animal species, and the processing used. To the best of our knowledge, there are few studies available in the literature on the content of L-carnitine in various food products. However, it has been found that the level of carnitine in processed meat is significantly lower compared to raw meat [47]. The results of this study showed that the content of L-carnitine in raw fermented sausages with the addition of tomato pomace was statistically lower compared to the control sample. Additionally, it was found that the results were lower compared to the results obtained by other researchers for other meat products. In raw fermented sausages from fallow deer and beef with the addition of acid whey, the content of L-carnitine ranged from 70.66 to 152.0 mg 100 g^−1^ of the product [48]. It was therefore many times higher compared to the results obtained in the current study. This could be due to the fact that tomato pomace contains sugars (e.g., glucose, fructose) that can be easily metabolized by lactic acid bacteria. This may lead to more intense fermentation and increased production of metabolites such as organic acids. This, in turn, may promote the degradation of L-carnitine, which is more susceptible to hydrolysis in an acidic environment. Additionally, despite the presence of antioxidants, tomato pomace may contain residues of plant enzymes (e.g., polyphenol oxidases). In the process of ripening meat products, they may generate reactive oxygen species (ROS), which oxidize L-carnitine. The addition of tomato pomace modifies the fermentation and ripening process, which reduces the stability and availability of L-carnitine in the final product [49].

## 4. Materials and Methods

### 4.1. Preparation of Experimental Material

In the experiment, raw fermented sausages containing 50 mg/kg of sodium nitrite and tomato pomace were studied. The raw materials were ham muscles from pork and backfat from Polish Large White fatteners. These materials were collected directly from a local slaughterhouse and delivered to the laboratory under refrigerated conditions within 48 h after slaughter. The sausage mixture was prepared in a ratio of 85% ham muscles to 15% backfat. Additional ingredients included 0.6% glucose, 2.8% curing mixture (sea salt + sodium nitrite), tomato pomace, and commercial starter cultures (Moguntia, Bessa START, Georgsmarienhütte, Germany). As stated by the manufacturer, the starter cultures contained *Staphylococcus xylosus* and *Pediococcus pentosaceus*. Commercial cultures were added in amounts of 30 g per 50 kg of meat and fat mixture. The meat and fat were ground using a grinder (KU2-3EK, Mesko-AGD, Skarżysko-Kamienna, Poland) with a plate having holes of 0.01 m diameter. The method of obtaining tomato pomace (TP) was presented in a previous publication [7]. The tomato pomace, consisting of seeds and peels, was ground just before use with a knife mill (Bosch TSM6A017C, München, Germany) to particles smaller than 0.3 mm in diameter. Three sausage variants were prepared: SK—control sample; STP 1.5%—sample with 1.5% tomato pomace; and STP 2.5%—sample with 2.5% tomato pomace. All ingredients were mixed using a universal machine (KU2-3EK, Mesko-AGD, Skarżysko-Kamienna, Poland) with an attached R4 agitator (100 rpm for 3 min), then filled into fibrous casings (ø 65 mm, Viskase Corporation, Chicago, IL, USA) using a manual sausage stuffer. The fermentation and drying process was carried out in fermentation chambers (ITALFROST-DE RIGO-GS, Pszczyna, Poland) for 30 days under controlled humidity and temperature conditions. The production conditions were as follows: Stage 1—temperature 20–22 °C, relative humidity 55–65%, for 3 days; Stage 2—temperature 14–16 °C, relative humidity 68–75%, for 3 days; Stage 3—temperature 13 °C, relative humidity 76%, for 13 days. Each post-production sample was analyzed three times for the content of volatile compounds and L-carnitine, and the sausages also underwent sensory analysis.

### 4.2. Analysis of Volatile Compounds

The analysis of volatile compounds was conducted using the SPME–gas chromatography–mass spectrometry (GC-MS) technique (Agilent Technologies, Santa Clara, CA, USA); following the method described by Pérez-Santaescolástica et al. [50], an SPME device (Supelco, Bellefonte, PA, USA) equipped with a 10 mm long fused silica fiber coated with a 50/30 μm DVD/CAR/PDMS layer was utilized. Before analysis, each sample was separately ground to obtain a homogeneous mixture. Extraction was performed by solid-phase microextraction. One gram of each sample was placed in a 20 mL vial with a cap. The samples were conditioned at 37 °C for 15 min to ensure uniform temperature. The extraction was then carried out by inserting the SPME fiber into the vial for 30 min. The fiber was then removed and transferred to the injection port of the GC-MS system. Chromatographic separation was performed using a 7890B chromatograph system (Agilent Technologies, Santa Clara, CA, USA) coupled with a 5977B mass spectrometer (Agilent Technologies). The capillary column used was DB-624 (length 30 m × 0.25 ID mm × 1.40 μm film thickness; J&W Scientific, Folsom, CA, USA). The injection port was operated in splitless mode, with the split valve opening after 2 min. Helium was used as the carrier gas at a constant flow rate of 1.2 mL/min (9.59 psi). The temperature program was as follows: initial temperature of 40 °C held for 10 min, then to 200 °C at 5 °C/min, followed by 250 °C at 20 °C/min with a final hold of 5 min; total scanning time was 50.8 min. Compounds were identified by comparing their mass spectra with those in the NIST05 library (National Institute of Standards and Technology, Gaithersburg, MD, USA) and/or by calculating the retention index relative to a series of reference alkanes (C5–C19) (for calculating the Kovats index, Supelco 44585-U, Bellefonte, PA, USA), with a fit factor of more than 85% taken into account. The results are reported in units of area (AU × 10^3^ g^−1^ sample).

### 4.3. Determination of L-Carnitine Content

The L-carnitine content was determined using the L-Carnitine Assay Kit MAK063 (Sigma-Aldrich, St. Louis, MO, USA), according to the procedure provided in the manufacturers technical bulletin. The results were expressed in milligrams of L-carnitine per 100 g of product.

### 4.4. Sensory Evaluation

The sensory quality was assessed using descriptive analysis with an unstructured linear graphical scale of 100 mm, converted to numerical values ranging from 0 to 10 conventional units (c.u.), according to ISO 13299:1998 (QDA) [51]. The sausages were sliced into approximately 2 mm thick pieces using an electric slicer and placed in transparent, odorless plastic containers with lids. Each sample was individually coded with three digits and presented randomly to minimize transfer effects. The samples were kept in the containers at room temperature (24 ± 1 °C) for 30 min before evaluation. Panelists assessed the samples in plastic containers with lids against a white background using evaluation cards. The parameters evaluated and their descriptors were as follows: meat color—from gray to very red; cross-sectional fat color—from gray to white; slice compactness—from low to very high; juiciness—from dry to very juicy; hardness—from low to very high; overall quality—from poor to very good. Additionally, the intensity of smell (meat, tomato, and other odors) and taste (meat, tomato, salty, sweet, and other flavors) were assessed. The evaluation was conducted by a 13-member panel from the Department of Meat Technology and Food Quality at the University of Life Sciences in Lublin. Consistent temperature, lighting, and elimination of distracting factors such as noise and unpleasant odors were maintained during the analysis.

### 4.5. Statistical Analysis

The data collected during this study were processed using Statistica software version 9.1 (StatSoft, Kraków, Poland) and presented as mean ± standard deviation. All measurements were performed with at least three replicates. The normality of the variable distributions in the study groups was assessed using the Shapiro–Wilk test. Data analysis was conducted using two-way analysis of variance (ANOVA). Differences between mean values were determined using Tukey’s test. A significance level of *p* < 0.05 was adopted, indicating the presence of statistically significant differences or relationships.

## 5. Conclusions

The results found in the present study confirmed that the tomato pomace had a significant effect on raw fermented sausages, since it contributed to the amount of volatile compounds as well as sensory attributes. The sausages with TP in the amount of 1.5% and 2.5% were characterized by positive sensory features, and their overall quality was rated on above 7 c.u. on a 10-point scale, similar to the control sausage. Significant differences were observed in the content of individual volatile substances included in the groups of volatile compounds (alcohols, aldehydes, hydrocarbons, and ketones) between the tested groups of sausages. The effect of the addition of tomato pomace in amounts of both 1.5% and 2.5% had a very similar effect on the assessed characteristics of raw fermented sausages.

## Figures and Tables

**Table 1:** Content of volatile compounds (AU × 10^3^ g^−1^ of sample) in raw fermented sausages.

Family	Compound	*m*/*z*	LRI	SK	TP 1.5%	TP 2.5%
Alcohols	Cyclobutanol	44	485	651.46 ± 963.43 ^a^	97.97 ± 79.75 ^a^	97.96 ± 25.55 ^a^
	1-Butanol, 3-methyl-	70	791	443.42 ± 329.91 ^a^	367.32 ± 317.50 ^a^	176.25 ± 8.52 ^a^
	1-Butanol, 2-methyl-	56	794	73.68 ± 45.63 ^a^	97.99 ± 55.07 ^a^	69.48 ± 5.86 ^a^
	1-Pentanol	70	828	212.54 ± 266.72 ^a^	191.96 ± 42.86 ^a^	233.03 ± 47.99 ^a^
	R-(−)-1,2-propanediol	45	855	15.90 ± 10.70 ^a^	25.97 ± 10.66 ^a^	30.99 ± 18.19 ^a^
	2,3-Butanediol, [R-(R*,R*)]-	45	889	2265.65 ± 1353.10 ^a^	1841.62 ± 1319.58 ^a^	3294.99 ± 1703.35 ^a^
	2-Heptanol	45	954	37.11 ± 11.83 ^a^	84.05 ± 1.62 ^b^	96.71 ± 6.42 ^b^
	6-Hepten-1-ol, 2-methyl-	95	1035	1.47 ± 0.16 ^a^	69.09 ± 5.02 ^b^	100.79 ± 11.00 ^c^
	Benzyl alcohol	108	1097	180.59 ± 75.09 ^a^	204.83 ± 51.33 ^a^	276.03 ± 65.96 ^a^
	Phenol, 2-methoxy-	124	1127	2.14 ± 0.12 ^a^	17.03 ± 3.59 ^b^	35.91 ± 6.61 ^c^
	Phenylethyl alcohol	91	1153	41.10 ± 14.02 ^a^	39.83 ± 16.33 ^a^	57.44 ± 10.66 ^a^
	Total alcohols			3925.05 ± 2343.66 ^a^	3037.66 ± 1851.25 ^a^	4469.58 ± 1663.33 ^a^
Aldehydes	Butanal, 3-methyl-	58	648	19.35 ± 15.07 ^a^	96.96 ± 20.35 ^b^	123.85 ± 18.65 ^b^
	Butanal, 2-methyl-	58	661	12.17 ± 9.75 ^a^	49.48 ± 6.73 ^b^	67.72 ± 4.50 ^b^
	Pentanal	58	714	61.87 ± 28.94 ^a^	129.75 ± 36.36 ^a^	224.99 ± 28.13 ^b^
	Hexanal	56	845	1433.80 ± 1535.38 ^a^	361.15 ± 92 ^a^	1270.75 ± 1525.15 ^a^
	Heptanal	70	949	131.27 ± 143.30 ^a^	52.12 ± 5.88 ^a^	97.73 ± 76.05 ^a^
	Methional	104	976	3.22 ± 0.27 ^a^	26.32 ± 5.90 ^b^	45.51 ± 2.57 ^c^
	Benzaldehyde	106	1019	69.44 ± 31.50 ^a^	285.28 ± 83.81 ^b^	319.00 ± 44.18 ^b^
	Benzeneacetaldehyde	91	1091	11.63 ± 10.13 ^a^	23.36 ± 3.34 ^ab^	35.13 ± 8.20 ^b^
	Nonanal	98	1117	49.04 ± 40.11 ^a^	35.64 ± 9.28 ^a^	43.54 ± 20.16 ^a^
	Total aldehydes		8022	1791.79 ± 1768.93 ^a^	1060.06 ± 223.18 ^a^	2228.23 ± 1715.36 ^a^
Carboxylic acids	Acetic acid	43	676	2809.05 ± 2367.05 ^a^	9387.22 ± 1206.98 ^b^	11,189.16 ± 1063.71 ^b^
	Butanoic acid	60	894	1201.48 ± 298.93 ^a^	2097.12 ± 296.77 ^b^	2767.75 ± 236.72 ^b^
	Hexanoic acid	60	1054	16.96 ± 15.26 ^a^	60.50 ± 17.13 ^b^	106.04 ± 9.27 ^c^
	Total carboxylic acids			4027.48 ± 2645.47 ^a^	11,544.84 ± 1503.33 ^b^	14,062.95 ± 1059.91 ^b^
Cyclic hydrocarbon	Spiro[2,4]hepta-4,6-diene	91	785	348.42 ± 85.95 ^a^	365.44 ± 17.02 ^a^	374.37 ± 13.70 ^a^
Cyclic hydrocarbon (aromatic)	Ethylbenzene	91	893	90.73 ± 12.60 ^a^	87.55 ± 1.32 ^a^	98.87 ± 13.16 a
	Benzene, 1,3-dimethyl-	91	902	230.49 ± 27.87 ^a^	248.28 ± 6.69 ^a^	276.66 ± 30.18 ^a^
	Total cyclic hydrocarbon (aromatic)			321.23 ± 38.84 ^a^	335.83 ± 7.49 ^a^	375.54 ± 42.50 ^a^
Esters	Acetic acid, methyl ester	74	523	28.26 ± 16.42 ^a^	140.38 ± 17.10 ^b^	152.31 ± 22.90 ^b^
	Ethyl Acetate	61	585	2003.42 ± 2461.30 ^a^	692.36 ± 162.74 ^a^	486.01 ± 42.40 ^a^
	Butanoic acid, methyl ester	74	737	24.44 ± 8.89 ^a^	115.80 ± 18.55 ^b^	122.91 ± 19.35 ^b^
	Butanoic acid, ethyl ester	88	834	3499.45 ± 5218.62 ^a^	385.67 ± 94.47 ^a^	273.30 ± 39.06 ^a^
	ETHYL (S)-(−)-LACTATE	45	873	8204.74 ± 12,720.72 ^a^	1093.88 ± 201.49 ^a^	863.88 ± 202.41 ^a^
	Hexanoic acid, methyl ester	74	959	16.26 ± 1.57 ^a^	43.64 ± 12.54 ^ab^	56.45 ± 14.68 ^b^
	Methyl salicylate	152	1194	0.00 ± 0.00 ^a^	21.73 ± 4.74 ^b^	43.34 ± 5.08 ^c^
	total esters			13,776.57 ± 20,369.95 ^a^	2493.46 ± 496.38 ^a^	1998.19 ± 327.29 ^a^
Ether	Dimethyl ether	45	503	16,476.45 ± 22,876 ^a^	2418.96 ± 567.22 ^a^	1699.87 ± 287.73 ^a^
Hydrocarbons	Pentane	72	500	31.37 ± 16.56 ^a^	35.48 ± 5.52 ^a^	30.95 ± 7.88 ^a^
	Pentane, 2-methyl-	71	527	134.57 ± 107.19 ^a^	184.86 ± 45.81 ^a^	115.90 ± 57.35 ^a^
	n-Hexane	57	545	191.83 ± 114.49 ^a^	222.48 ± 24.90 ^a^	341.19 ± 166.35 ^a^
	Butane, 2,2,3,3-tetramethyl-	57	640	68.25 ± 56.71 ^a^	96.91 ± 27.46 ^a^	80.45 ± 11.05 ^a^
	Octane	85	800	407.91 ± 166.03 ^a^	242.65 ± 71.33 ^a^	189.97 ± 41.99 ^a^
	Heptane, 2,2,4,6,6-pentamethyl-	57	997	195.81 ± 71.28 ^a^	235.60 ± 26.79 ^a^	575.96 ± 274.02 ^a^
	Decane	57	1000	49.91 ± 12.61 ^a^	79.68 ± 2.12 ^a^	54.49 ± 16.99 ^a^
	2-Undecene, 9-methyl-, (Z)-	98	1099	20.24 ± 6.79 ^a^	28.86 ± 3.70 ^a^	24.14 ± 6.54 ^a^
	Total hydrocarbons			1099.91 ± 470.24 ^a^	1126.53 ± 24.41 ^a^	1413.05 ± 330.77 ^a^
Hydrocarbons (cyclic)	1,3,5,7-Cyclooctatetraene	104	930	230.49 ± 27.87 ^a^	248.28 ± 6.69 ^a^	276.66 ± 30.18 ^a^
	Cyclopropane, propyl-	56	931	32.64 ± 6.32 ^a^	32.67 ± 4.27 ^a^	41.34 ± 5.15 ^a^
	Total hydrocarbons (cyclic)			263.13 ± 33.76 ^a^	280.95 ± 4.01 ^a^	318.01 ± 35.30 ^a^
Ketones	Acetone	58	513	93.21 ± 70.14 ^a^	202.20 ± 8.26 ^b^	243.62 ± 19.43 ^b^
	2-Butanone	72	581	65.00 ± 53.13 ^a^	164.42 ± 15.24 ^b^	163.61 ± 8.69 ^b^
	Acetoin	45	774	14,992.04 ± 19,377.11 ^a^	2437.17 ± 3742.92 ^a^	731.88 ± 547.26 ^a^
	1-Hepten-3-one	70	900	3.33 ± 1.24 ^a^	14.79 ± 1.99 ^b^	14.86 ± 1.41 ^b^
	5-Hepten-2-one, 6-methyl-	108	1029	4.17 ± 1.49 ^a^	211.77 ± 21.78 ^b^	308.77 ± 80.64 ^b^
	Acetophenone	105	1108	50.19 ± 23.45 ^a^	51.57 ± 7.59 ^a^	53.73 ± 14.39 ^a^
	2-Nonanone	58	1110	32.19 ± 6.18 ^a^	73.38 ± 11.94 ^b^	84.97 ± 6.75 ^b^
	Total ketones			15,240.12 ± 19,415.14 ^a^	3155.30 ± 3776.26 ^a^	1601.43 ± 456.67 ^a^
Terpenoids	Prenol	71	837	461.29 ± 134.28 ^a^	430.10 ± 50.07 ^a^	391.75 ± 57.73 ^a^
	3-Carene	93	951	143.79 ± 85.24 ^a^	184.54 ± 59.62 ^a^	275.08 ± 94.93 ^a^
	Bicyclo[3.1.0]hex-2-ene, 4-methyl-1-(1-methylethyl)-	93	995	34.74 ± 19.59 ^a^	44.73 ± 15.69 ^a^	65.21 ± 20.96 ^a^
	Total terpenoids			639.82 ± 109.97 ^a^	659.36 ± 118.74 ^a^	732.03 ± 64.37 ^a^
Others	2-Hydrazinoethanol	45	517	16,086.76 ± 23,160.04 ^a^	268.46 ± 38.95 ^a^	238.74 ± 68.00 ^a^
	Sulphuric acid dibutyl ester	56	535	47.87 ± 34.37 ^a^	66.59 ± 10.74 ^a^	39.83 ± 18.02 ^a^
	Disulfide, dimethyl	94	764	30.65 ± 27.99 ^a^	313.95 ± 210.47 ^a^	376.11 ± 168.18 ^a^
	Dimethyl trisulfide	126	1008	21.69 ± 26.84 ^a^	143.56 ± 132.11 ^a^	129.88 ± 67.15 ^a^
	Furfurylmethylamphetamine	81	1009	93.13 ± 90.99 ^a^	37.88 ± 4.67 ^a^	67.68 ± 41.03 ^a^
	Dimethyl sulfone	79	1050	187.97 ± 77.66 ^a^	330.27 ± 151.09 ^a^	560.07 ± 359.20 ^a^
	2-Isobutylthiazole	99	1058	66.33 ± 109.06 ^a^	225.28 ± 68.56 ^ab^	283.74 ± 12.21 ^b^
	2,4-Imidazolidinedione 1-methyl-	114	1295	9.00 ± 9.01 ^a^	18.43 ± 11.13 ^a^	32.98 ± 16.94 ^a^
	Total others			16,543.40 ± 23,018.47 ^a^	1404.43 ± 269.50 ^a^	1729.04 ± 462.02 ^a^
	Total compounds			74,453.35 ± 55,416.33 ^a^	27,882.83 ± 5596.69 ^a^	31,002.29 ± 1301.87 ^a^

SK—control sample; TP 1.5%—sample with 1.5% addition of tomato pomace; TP 2.5%—sample with 2.5% addition of tomato pomace. Means with different lowercase letters (a–c) differ significantly (*p* < 0.05). LRI—linear retention index; *m*/*z*—mass-to-charge ratio.

**Table 2:** L-carnitine content in raw fermented sausages.

Parameter	SK	TP 1.5%	TP 2.5%
L-carnitine (mg 100 g^−1^ of product)	2.51 ± 0.18 ^b^	1.60 ± 0.10 ^a^	1.39 ± 0.04 ^a^

SK—control sample; TP 1.5%—sample with 1.5% addition of tomato pomace; TP 2.5%—sample with 2.5% addition of tomato pomace. Means with different lowercase letters (a, b) differ significantly (*p* < 0.05).

**Table 3:** Sensory parameters of raw fermented sausages.

Parameter	SK	TP 1.5%	TP 2.5%
Color of meat in cross-section	6.02 ± 0.982 ^a^	8.38 ± 1.02 ^b^	9.03 ± 0.98 ^b^
Color of fat in cross-section	8.42 ± 1.11 ^a^	7.592 ± 1.58 ^a^	7.58 ± 2.10 ^a^
Juiciness	5.27 ± 0.96 ^a^	5.90 ± 1.24 ^a^	5.98 ± 1.17 ^a^
Hardness	4.42 ± 1.16 ^a^	3.84 ± 1.62 ^a^	4.15 ± 1.89 ^a^
Intensity of meat odor	6.52 ± 1.74 ^b^	2.84 ± 1.77 ^a^	2.91 ± 2.40 ^a^
Intensity of tomato odor	0.78 ± 1.59 ^a^	4.77 ± 2.21 ^b^	5.89 ± 1.64 ^b^
The intensity of other odor	1.59 ± 1.52 ^a^	1.65 ± 0.97 ^a^	1.72 ± 1.56 ^a^
Intensity of meat flavor	7.67 ± 1.32 ^b^	3.91 ± 2.32 ^a^	3.59 ± 1.93 ^a^
Intensity of tomato flavor	0.92 ± 1.70 ^a^	5.39 ± 2.76 ^b^	6.76 ± 1.34 ^b^
Intensity of salty flavor	5.09 ± 1.82 ^a^	4.52 ± 2.52 ^a^	4.32 ± 2.30 ^a^
Intensity of sweet flavor	0.99 ± 0.87 ^a^	1.88 ± 2.27 ^a^	2.19 ± 2.39 ^a^
Intensity of other flavor	0.73 ± 1.10 ^a^	1.35 ± 1.92 ^a^	1.91 ± 2.16 ^a^
Overall quality	7.45 ± 1.88 ^a^	7.85 ± 1.46 ^a^	8.03 ± 0.93 ^a^

SK—control sample; TP 1.5%—sample with 1.5% addition of tomato pomace; TP 2.5%—sample with 2.5% addition of tomato pomace. Means with different lowercase letters (a, b) differ significantly (*p* < 0.05).

## Data Availability

The original contributions presented in the study are included in the article, further inquiries can be directed to the corresponding author.
